# Supplementary material for: Microenvironment-derived factors driving metastatic plasticity in melanoma
Source: Nat Commun. 2017 Feb 9;8:14343. doi: 10.1038/ncomms14343 (PMC5309794; doi:10.1038/ncomms14343)
Supplement: Supplementary Information — Supplementary Figures [file ncomms14343-s1.pdf]

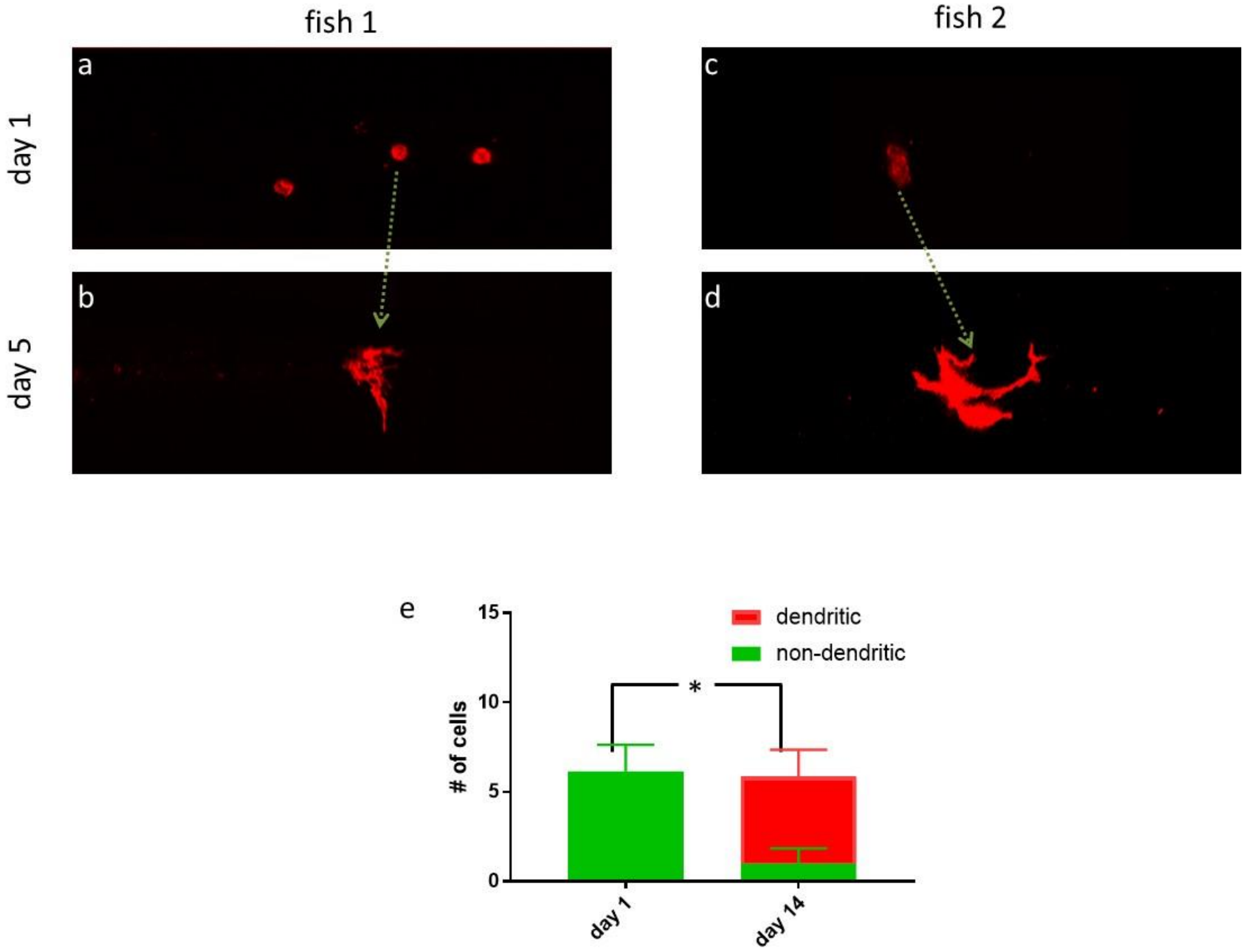

Supplementary Figure 1: Engraftment of metastatic cells is associated with a phenotype switch which yields highly dendritic cells within 5 days post transplant. A ZMEL1-derivative cell line containing a palmitylated tdTomato cassette was transplanted into 2dpf zebrafish embryos (n=10 fish) and these fish were subject to confocal imaging on day of transplant or 5 days post transplant. (a,b): This is one represeantative fish with cells in red shown on either day 1 or day 5 post transplant. The green arrow indicates how the morphology of that one cell changes over this period of time, acquiring a highly dendritic branched appearance. The fate of the other 2 cells seen on day 1 are not known but likely underwent apoptosis as is commonly seen after transplantation (c,d): A second representative fish showing the same pattern of morphological change over this time period. (e) Quantification of dendricity after transplant. Individual cells were counted and a dendritic cell was defined as any cell with at least 2 projections leading away from the cell body. At day 1, 100% of the cells had a rounded appearance with no evidence of dendricity, in contrast to cells at day 5 where 82% of the cells now had at least 2 dendrites (*,$p<0.05$, t-test).

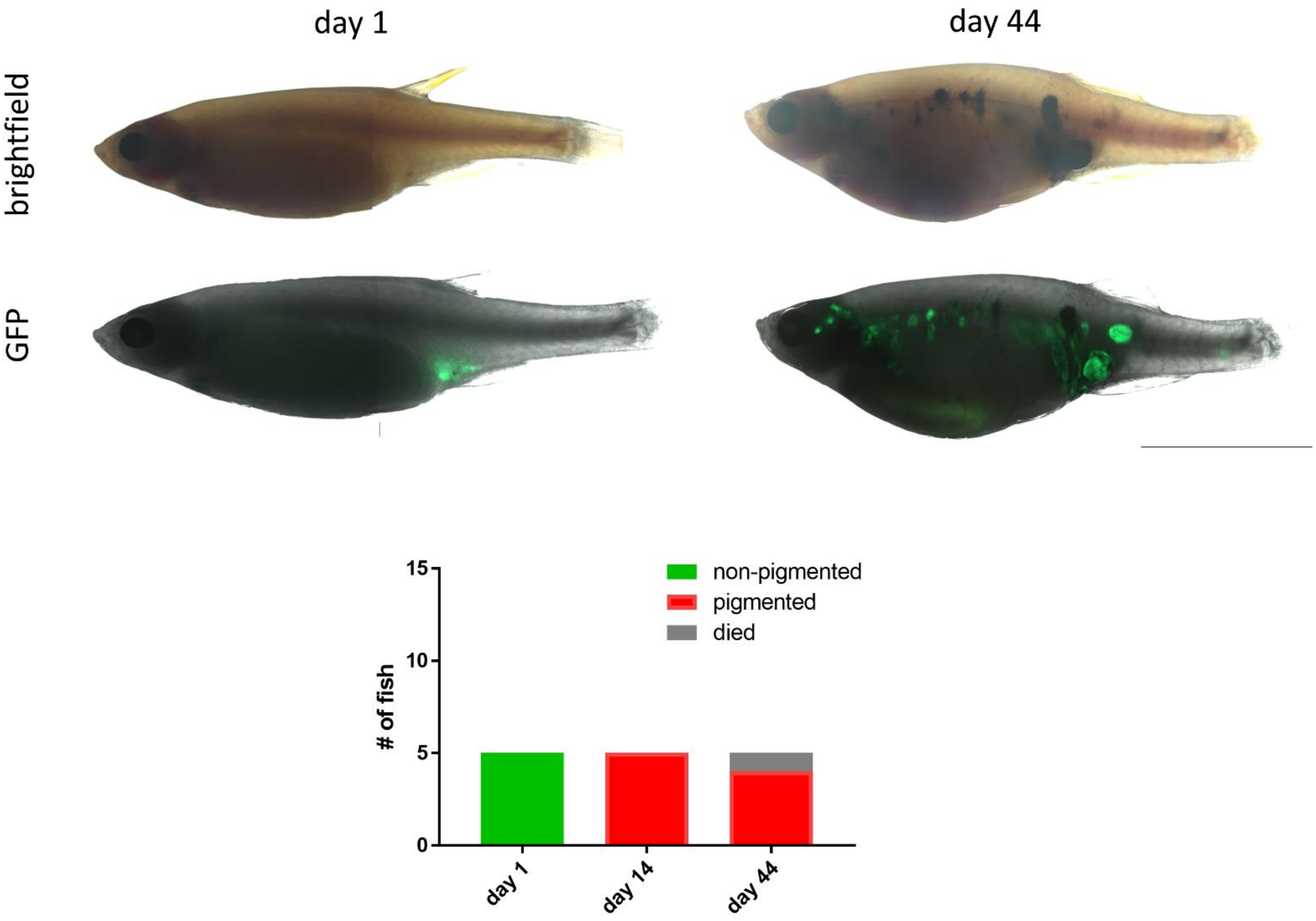

Supplementary Figure 2: Transplantation of the ZCREST cell line recapitulates the phenotype switching seen with the ZMEL1 line. A total of 5 fish were transplanted with the ZCREST-GFP line in a manner identical to that used for the ZMEL1 line, as described in the methods section. Five fish were imaged from 1-44 days post transplant and showed GFP positive tumors that became pigmented by 14-44 days post transplant.

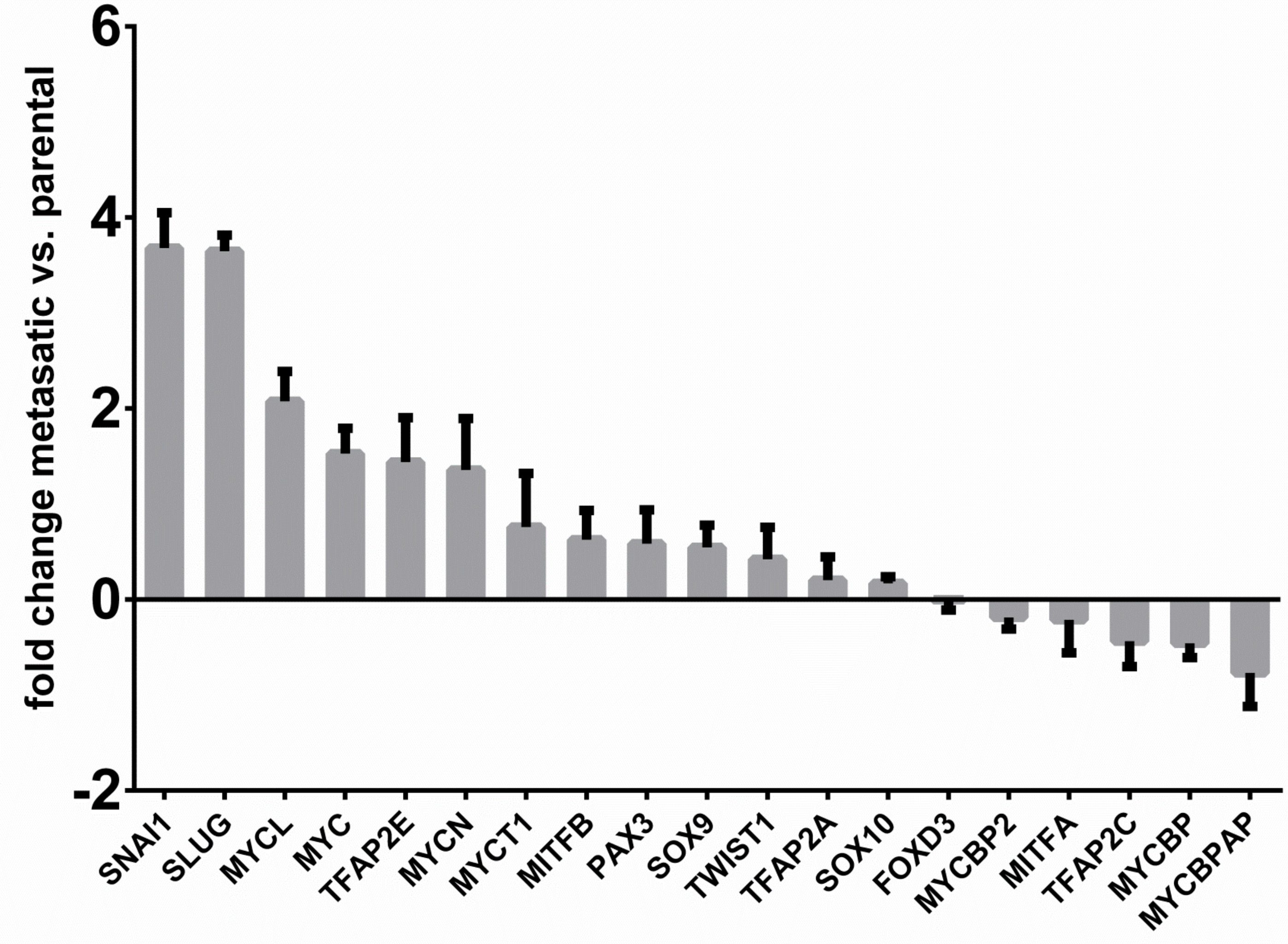

Supplementary Figure 3: Transcription factors altered in the disseminated vs. parental ZMEL1 cells, showing an upregulation of proliferation-related factors such as MYCL and MYC, along with a modest increase in MITFB (one of the two zebrafish isoforms of MITF).

Cox Proportional Hazards Analysis in TCGA Melanoma for Stage 1

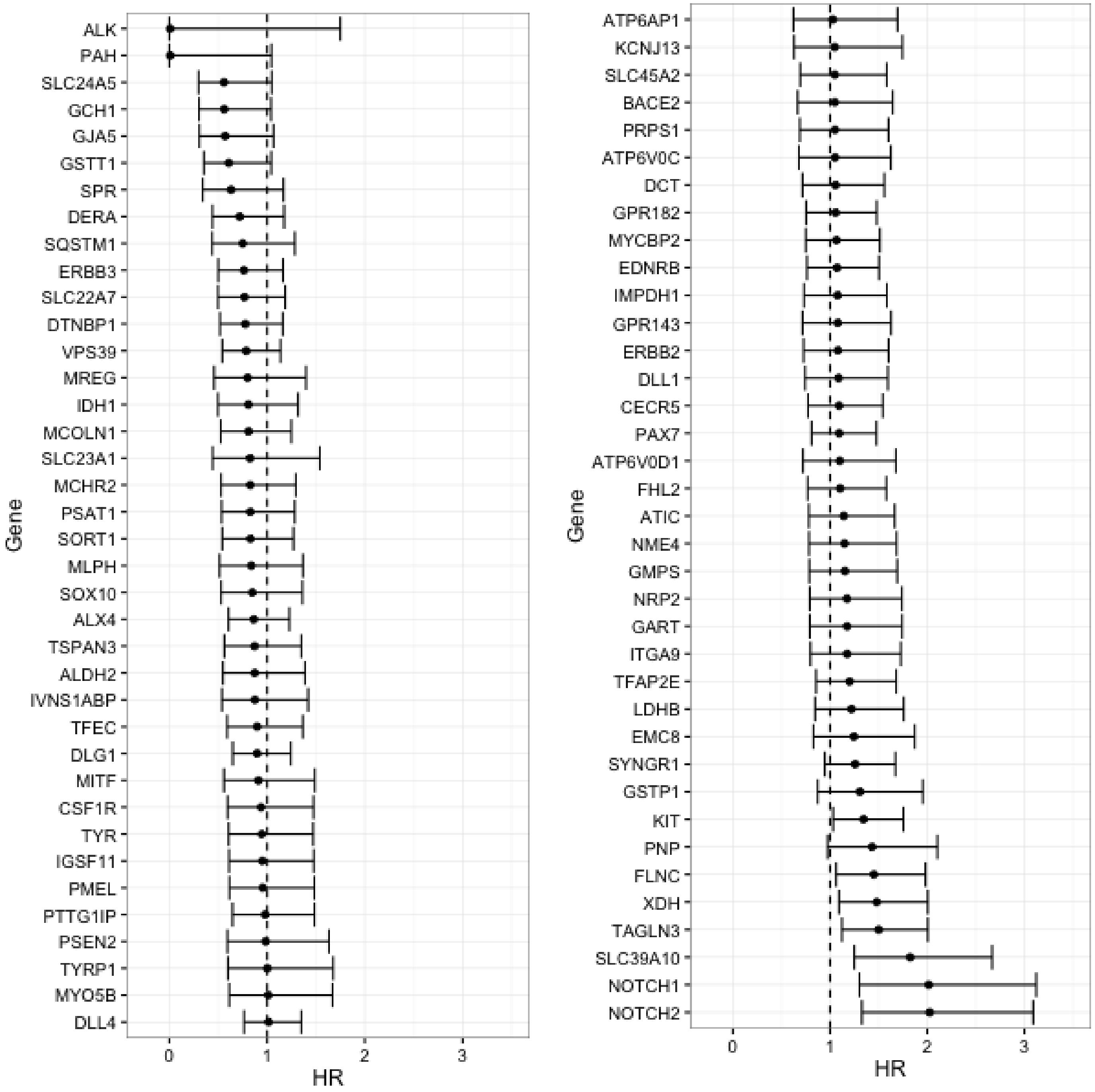

Supplementary Figure 4: Cox-Proportional Hazard Ratio plots for melanocyte differentiation genes in Stage I patients of the TCGA cohort. None of the canonical differentiation associated genes (i.e. PMEL, TYR) affect prognosis in this cohort.

Cox Proportional Hazards Analysis in TCGA Melanoma for Stage 3 and 4

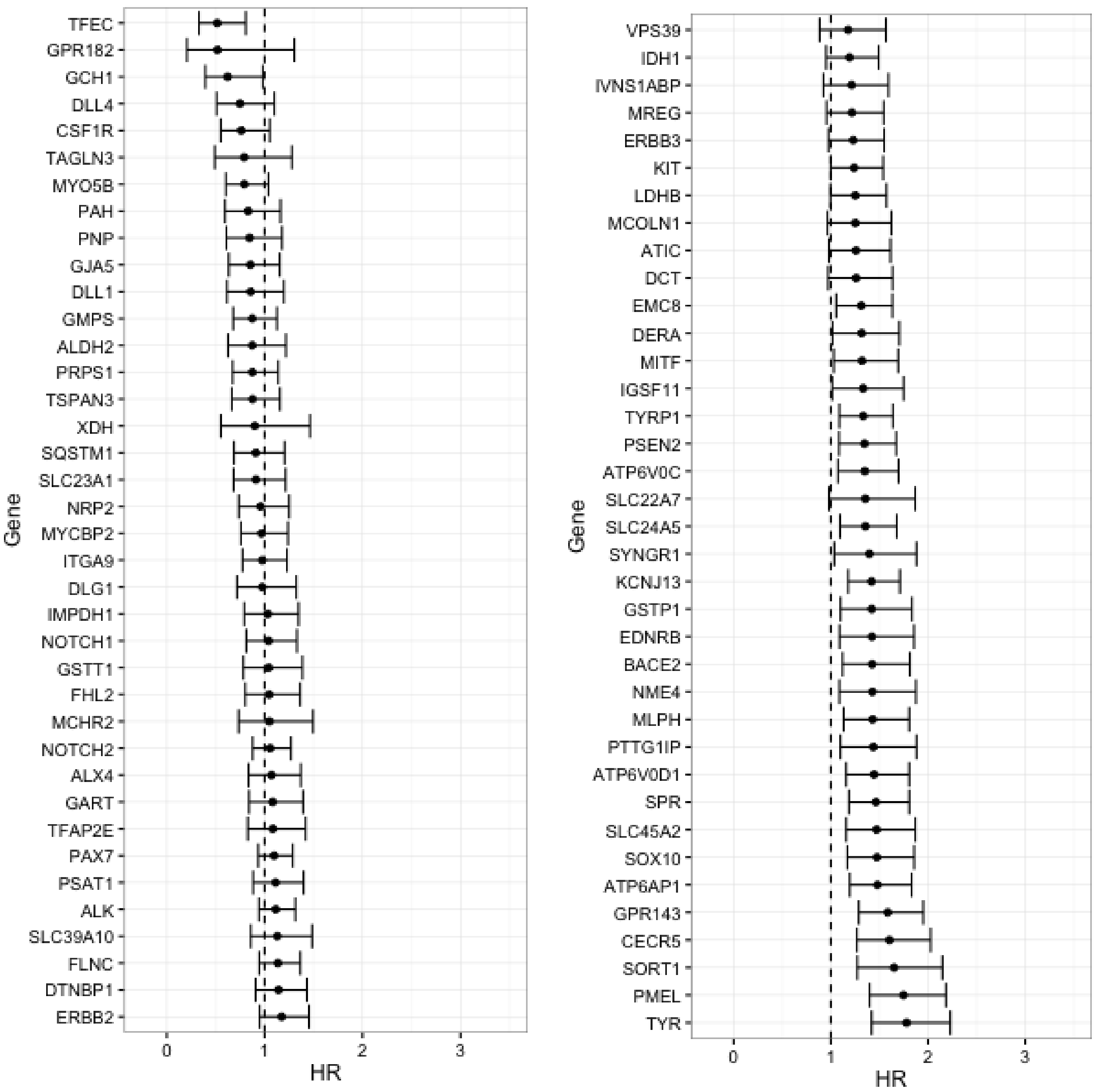

Supplementary Figure 5: Cox-Proportional Hazard Ratio plots for melanocyte differentiation genes in Stage III/IV patients of the TCGA cohort. In contrast to the stage I patients, high expression of a cluster of genes strongly associated with melanocyte differentation (i.e. PMEL, TYR) are negatively associated with survival in this cohort.

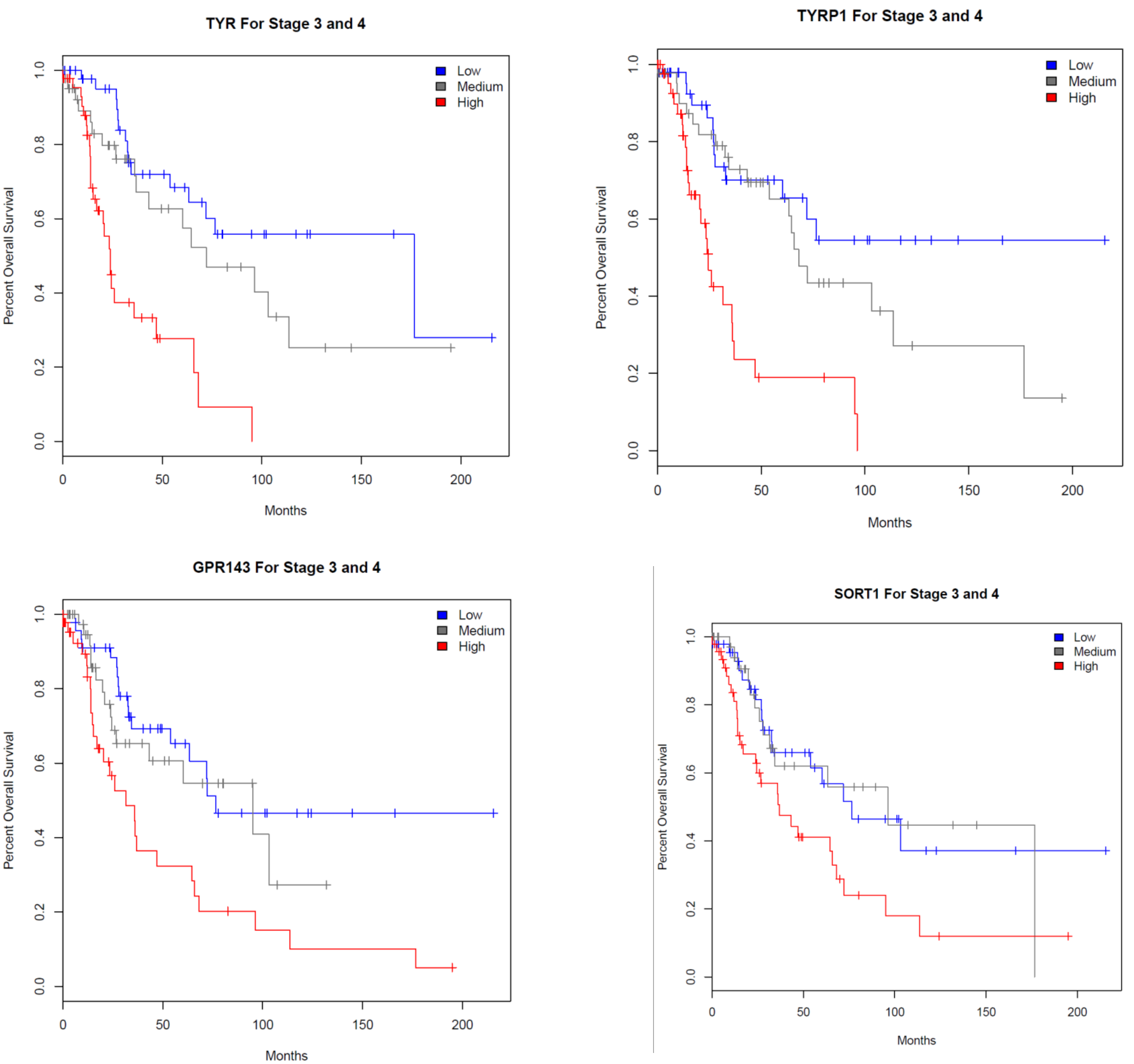

Supplementary Figure 6: Kaplan-Meier survival curves for 4 representative melanocyte differentiation genes in stage III/IV patients. For all 4 of these examples, higher expression is associated with significantly worse survival in this cohort.

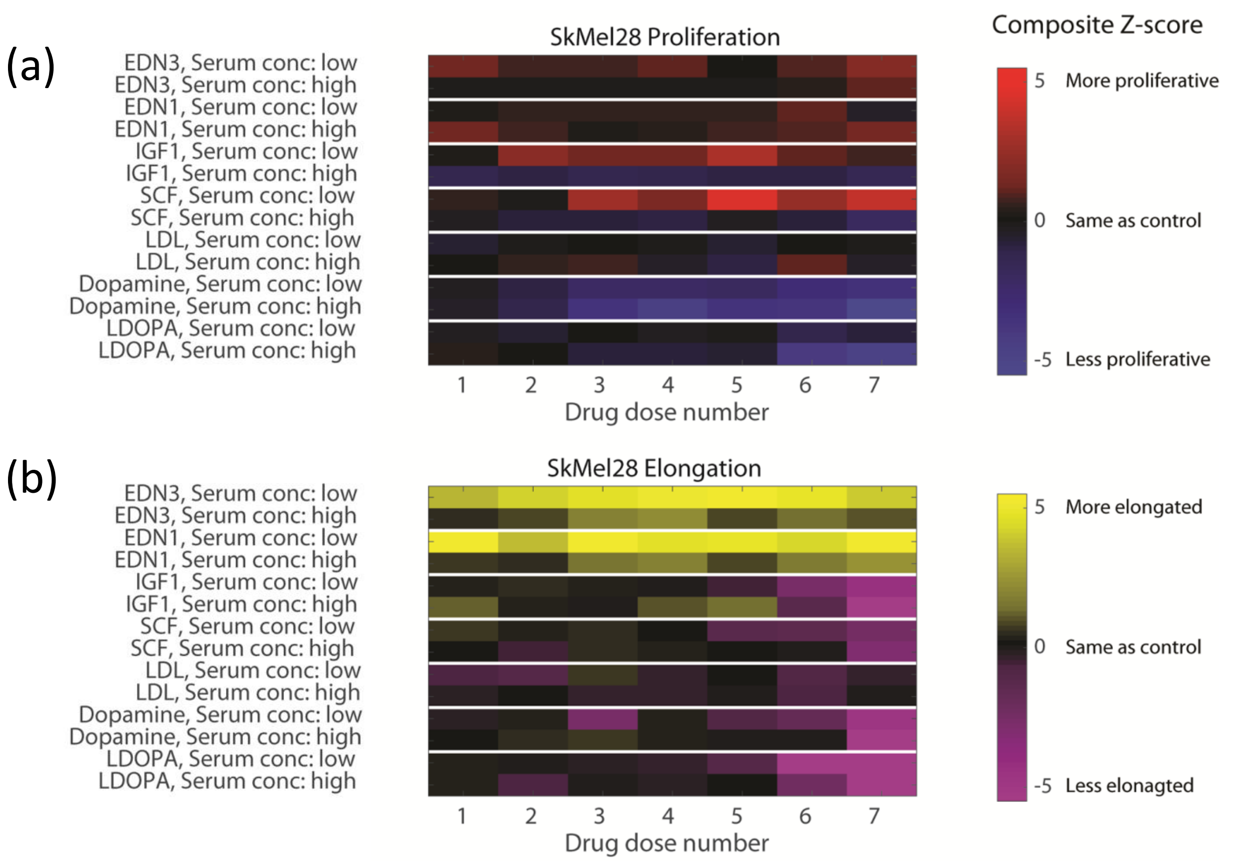

Supplementary Figure 7: (a) Heatmaps showing the effect of the various agonists on proliferation tested in either low or high serum conditions in SKMel28 human melanoma cells. Red indicates increased proliferation whereas blue indicates decreased proliferation (compared to DMSO control). (b) Heatmaps showing the effect of the agonists on cell elongation, which is a reflection of melanoma differentiation, in SKMel28 melanoma cells. Yellow indicates increased elongation, while purple indicates decreased proliferation.

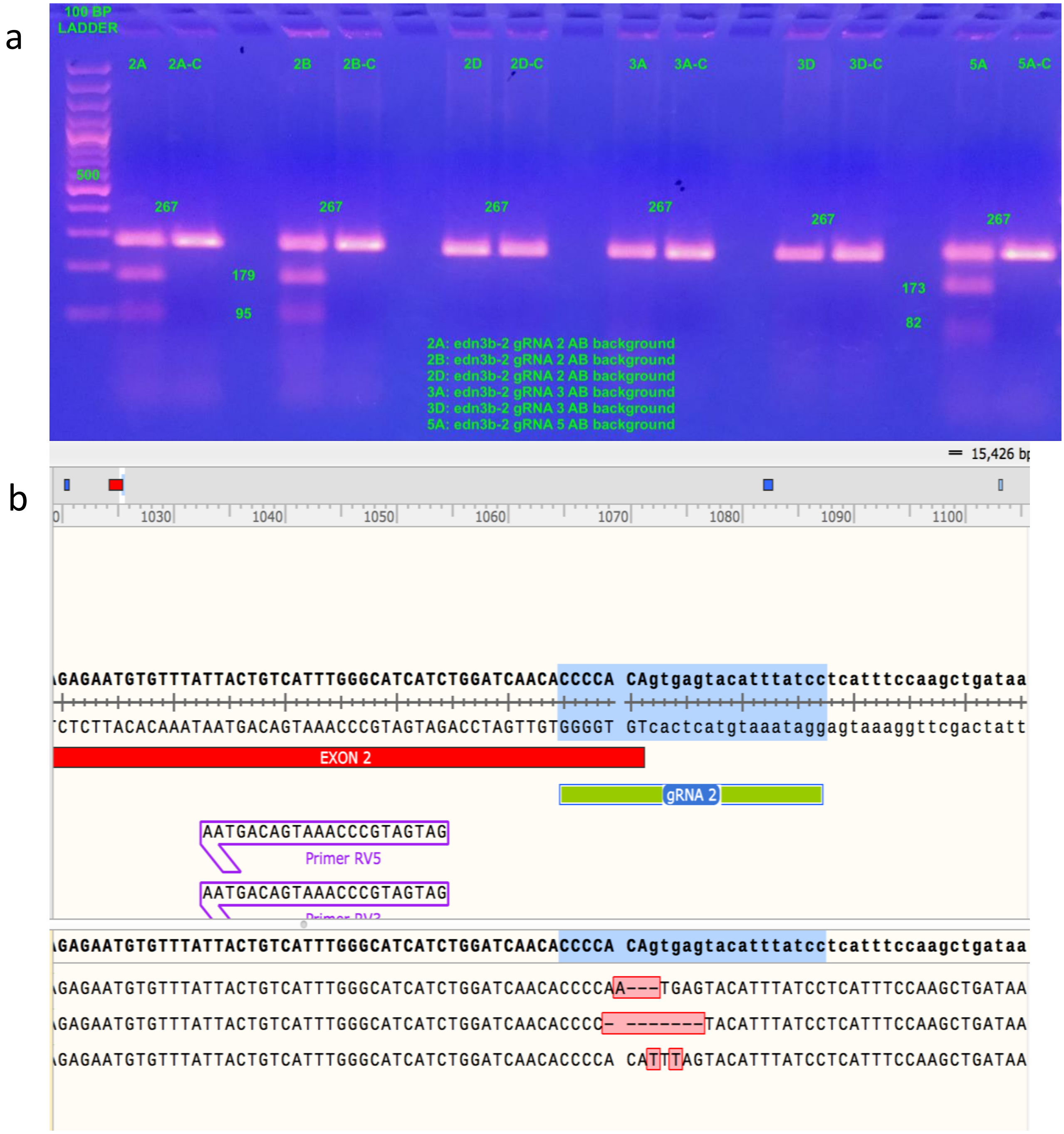

Supplementary Figure 8: Generation of a CRISPR mutant to EDN3B. (a) T7 endonuclease gel for 6 founder pairs injected with the EDN3B gRNA2 (sequence below). Pairs 1, 2 and 6 all showed the expected cutting pattern when compared to the control PCR product. (b) TOPO cloning of genomic DNA around the cut site shows a range of induced mutations, including a 4bp indel, an 8bp deletion, and a 2bp indel, all of which are predicted to lead to a loss of function after exon 2.

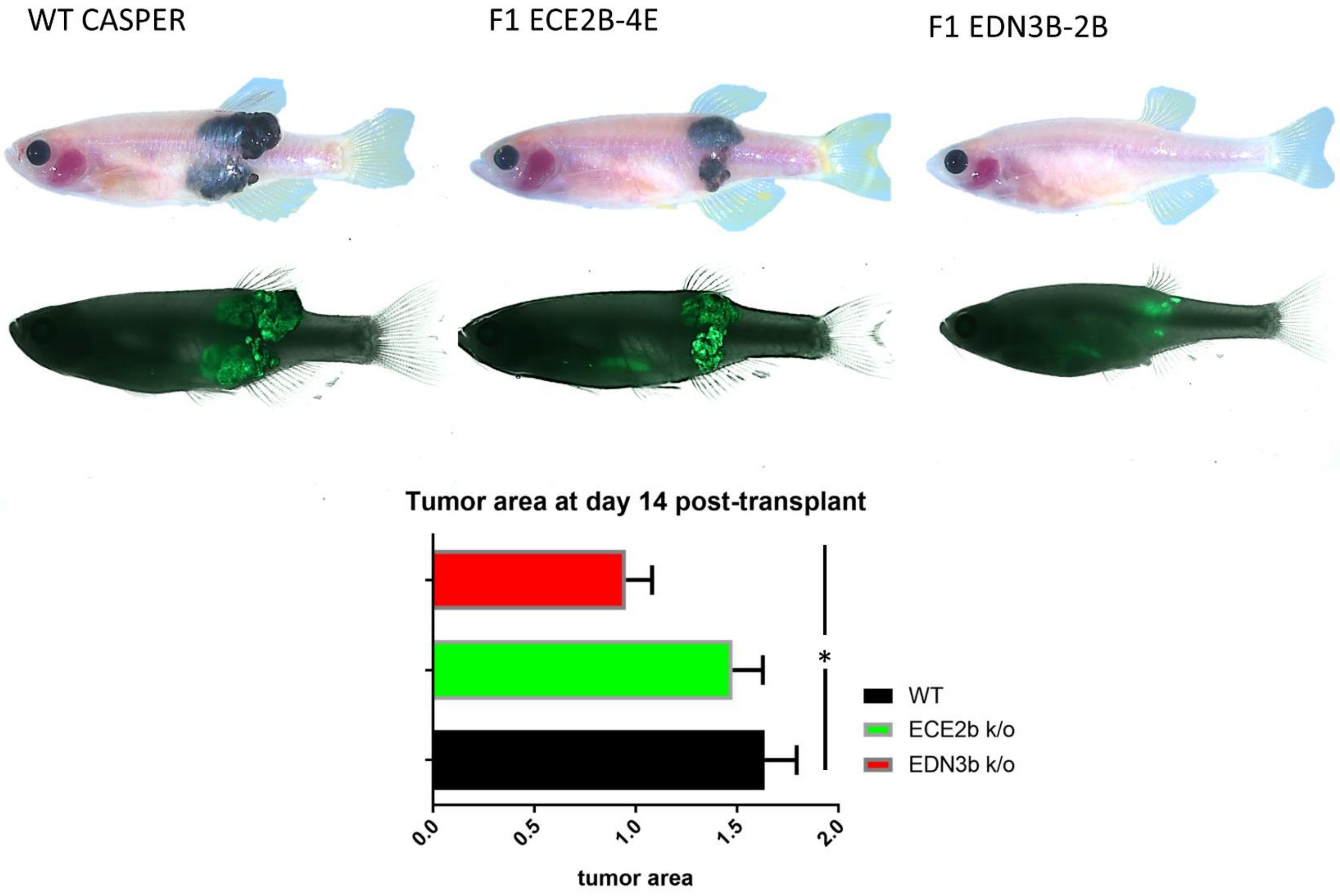

Supplementary Figure 9: Transplantation of ZMEL1-GFP cells into the ECE2b or EDN3b backgrounds in the *casper* strain (n=5 *casper,* n=6 EDN3b, n=7 ECE2b). Similar to what is seen in the WT background, microenvironmental deficiency of these factors leads to smaller tumors that are less pigmented, the quantification of which is shown below. *,p<0.05, ANOVA.

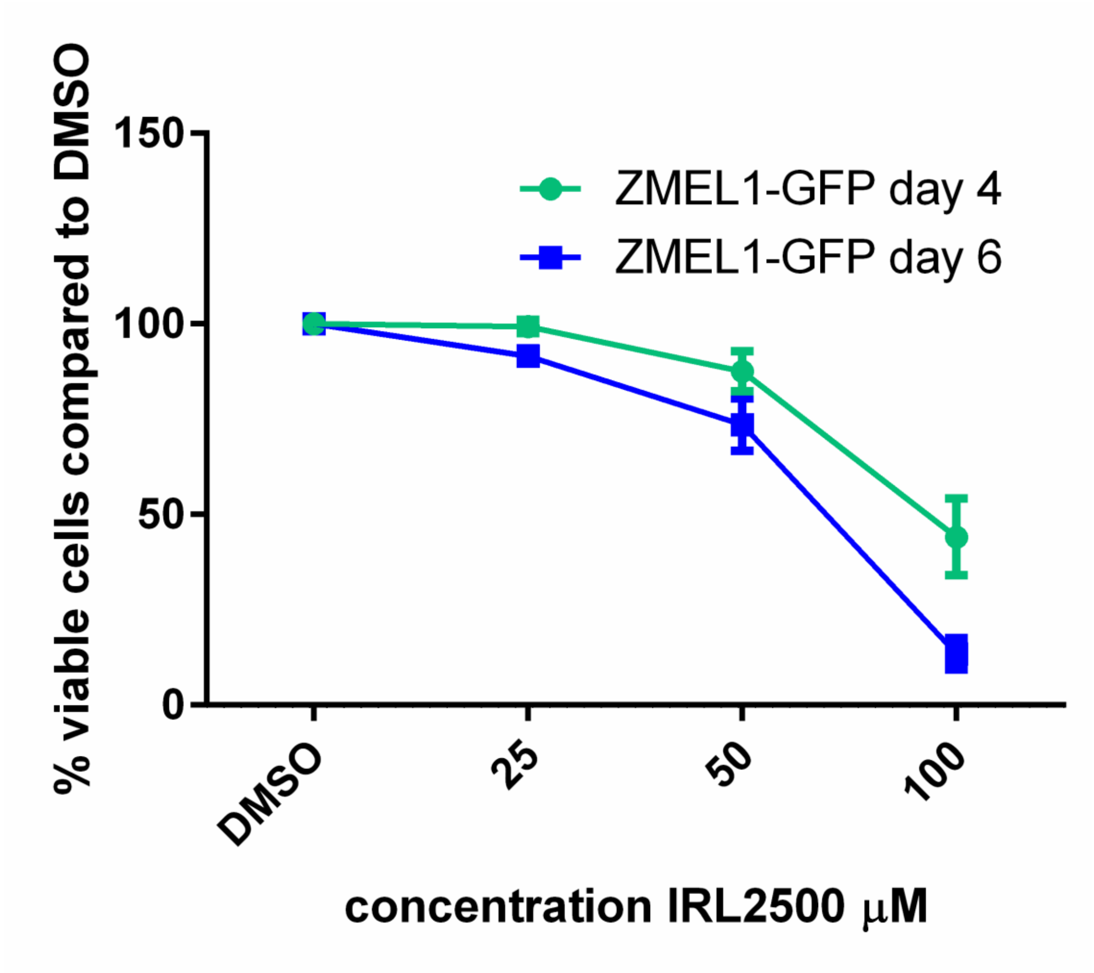

Supplementary Figure 10: Cell viability to the indicated doses of IRL2500, an EDNRB small molecule antagonist. Viability was measured at day 4 or day 6 of treatment using Cell-TiterGlo in ZMEL1 melanoma cells, and shows a decrease in viability only at 50-100 μM.
